# Attracting STEM Talent: Do STEM Students Prefer Traditional or Work/Life-Interaction Labs?

**DOI:** 10.1371/journal.pone.0089801

**Published:** 2014-02-27

**Authors:** William C. DeFraine, Wendy M. Williams, Stephen J. Ceci

**Affiliations:** Department of Human Development, Cornell University, Ithaca, New York, United States of America; Boston College, United States of America

## Abstract

The demand for employees trained in science, technology, engineering, and mathematics (STEM) fields continues to increase, yet the number of Millennial students pursuing STEM is not keeping pace. We evaluated whether this shortfall is associated with Millennials' preference for flexibility and work/life-interaction in their careers-a preference that may be inconsistent with the traditional idea of a science career endorsed by many lab directors. Two contrasting approaches to running STEM labs and training students were explored, and we created a lab recruitment video depicting each. The *work-focused* video emphasized the traditional notions of a science lab, characterized by long work hours and a focus on individual achievement and conducting research above all else. In contrast, the *work/life-interaction-focused* video emphasized a more progressive view – lack of demarcation between work and non-work lives, flexible hours, and group achievement. In Study 1, 40 professors rated the videos, and the results confirmed that the two lab types reflected meaningful real-world differences in training approaches. In Study 2, we recruited 53 current and prospective graduate students in STEM fields who displayed high math-identification and a commitment to science careers. In a between-subjects design, they watched one of the two lab-recruitment videos, and then reported their anticipated *sense of belonging* to and *desire to participate* in the lab depicted in the video. Very large effects were observed on both primary measures: Participants who watched the *work/life-interaction-focused* video reported a greater *sense of belonging* to (*d* = 1.49) and *desire to participate* in (*d* = 1.33) the lab, relative to participants who watched the *work-focused* video. These results suggest Millennials possess a strong desire for work/life-interaction, which runs counter to the traditional lab-training model endorsed by many lab directors. We discuss implications of these findings for STEM recruitment.

## Introduction

The U.S. faces a shortage of high-level STEM (Science, Technology, Engineering, and Mathematics) talent. There are not enough qualified college graduates to fill government STEM jobs [1], and an increasing number of students are bypassing academic jobs in STEM disciplines [2]. In addition, the rate of STEM degrees granted to domestic students is declining [3]. Overall, the demand for employees in STEM is increasing, yet the number of students pursuing STEM careers is not keeping pace. There are multiple causes of this problem [2], [3]. One reason is that the youngest cohorts of STEM majors, those born between late 1982 and 2000 and entering the workforce between 2005 and 2022-so-called *Millennials*-differ from their predecessors in attitudes, values, and preferences about the nature of the workplace and specifically, the optimal level of work/life-interaction [4], [5].

The term *work/life-interaction*, or *work/family-interaction*, refers to the degree to which work lives and non-work or family lives are integrated, rather than confined to their respective work versus non-work domains [5]. The change in norms that occurred in the latter part of the 20th century, from families with stay-at-home moms to those with two working parents, led to the concept of work/life-balance, in which parents carefully balance their time between responsibilities to their careers and to their families [5]. The limited time resources available in a given day makes the attempt to balance work and family needs difficult, and often results in one set of needs being shortchanged when the other set demands more time and attention [5]. Thus, Halpern suggested that the unsustainability of the work/life-balance concept calls for a re-evaluation of how we view work, and more specifically, that the degree of work/life-interaction should be maximized for the benefit of employers, employees, and their families [5]. At its core, the work/life-interaction concept entails flexible working environments in which employees' non-work needs and responsibilities are recognized and accommodated by employers [5], [6]. An example would be allowing employees to adjust their work hours so they can pick their children up from school or day care. However, the work/life-interaction concept also entails collaborative work environments wherein employees rely on one another, and group-achievement is emphasized. A collaborative environment allows an employer to provide flexibility to its employees without sacrificing the productivity of the company itself. When a group of employees collaborate on a project, it ensures that work on the project will continue if and when one member leaves the workplace early to attend to non-work needs. Thus, both flexibility and collaboration are integral to achieving work/life-interaction, and to avoiding the pitfalls inherent in the work/life-balance concept [5], [6].

Evidence suggests young STEM employees today prefer intertwined professional and personal lives within collaborative and flexible work environments. Surveys repeatedly indicate that Millennials desire greater work/life-interaction in their jobs than did previous generations [4], [7]. Many view STEM careers as imbalanced toward work without regard for broader life demands, and thus opt for non-STEM careers [2]. Two interesting aspects of this cultural shift are that: a) the desire for greater work/life-interaction has long been viewed as a women's issue [2], though increasingly an economic justification has been made for employers to provide such interaction for employees of both sexes [6], [8], and b) the work/life-interaction view contrasts starkly with the traditional notion of the solitary, goal-oriented lifestyle of a scientist.

Some high-profile technology employers (e.g., Evernote, Google, Cisco, et al.) have embraced Millennials' desire to lead integrated, so-called “360-degree lives,” embedded in an always-on corporate culture. To attract the best talent, these employers integrate employees' non-work needs with their jobs by providing flexible working conditions and a wide range of services (e.g., on-site health clubs, hair stylists, cafeteria meals for employees' families on late-working nights, automobile oil changes) that blur the traditional boundaries between work and non-work [9].

In contrast, STEM fields within academia still operate largely the way they did many decades ago: A successful career requires long hours in the lab in addition to teaching, and in general, dedication to one's work above all else – including family life. This “traditional” model was sensible at a time when the vast majority of academics and Ph.D. recipients were men who were the sole income-earners in their families [2], [10]. Today, however, women represent a steadily increasing proportion of STEM doctorate recipients. In many fields (e.g., biology, medicine, veterinary science), the number of doctoral degrees granted to women is keeping pace with or outpacing the number granted to men [10]. But many women have found it difficult to fulfill the demands of completing a doctorate and pursuing a tenure-track professorship at a time in life when they also aspire to have children [11]. Current research indicates that men in the Millennial generation are also expressing a preference for family-friendliness and flexibility in their careers [2]. Simply put, the traditional academic culture may not be optimally structured to accommodate changing demographics and women who want to raise families, much less a generational change in values and preferences toward work.

It is possible that the decline in domestic STEM Ph.D.s can be partly attributed to college students' exposure to traditional academic life in STEM fields, which inadvertently skews their view of STEM careers as a whole. Many current senior science lab directors-the “Boomer” generation-were socialized in an era when life revolved around work and boundaries between work and non-work were clear [6]. However, the current cultural shift in values and preferences toward work calls for an examination of how we recruit (or fail to recruit) the most talented young people to join the scientific workforce today. Do typical recruitment and training strategies used by traditional Boomer lab directors appeal to today's talented young STEM students? Or, are we unwittingly discouraging these students from pursuing STEM careers because of these traditional views possessed by lab directors? If so, are we discouraging women more than men because of their presumed greater desire for work/life-interaction, or do Millennials of both genders equally prefer the same type of training experience?

In this study we explored whether preference for greater work/life-interaction influences talented STEM students' decisions to join a hypothetical lab for graduate training. To examine this issue, we first created two lab recruitment videos that differed in their portrayal of the graduate training experience in the lab: one video reflected the traditional notions of science in academia that focuses on work above all else, and the second video reflected a more modern perspective highlighting the principles of work/life-interaction. In Study 1, we recruited a sample of current faculty members to validate the two videos in terms of the videos' accuracy in portraying real-world labs and conceptually distinct approaches to graduate training. Each faculty member was asked to rate the similarity of each video to the way he or she had been trained as a graduate student, and to the way he or she trains graduate students today. In Study 2, each participant in a sample of current STEM students watched one of the two recruitment videos, and provided ratings of how likely he or she would be to join and how comfortable he or she would feel being a part of the lab portrayed in the video. We also evaluated whether the two videos differentially affected students' commitment to pursuing a science career.

## Study 1

We hypothesized that a traditional work-focused approach to training graduate students, on the one hand, and an approach that incorporates more work/life-interaction, on the other hand, are two distinct types of training methods used in real-world research labs. Also, we hypothesized that the way a person was trained as a graduate student influences the way he or she later trains graduate students. That is, lab directors train graduate students in a way similar to the way they themselves were trained.

### Method

#### Ethics Statement

Cornell University's Institutional Review Board for Human Participants approved the protocol for this study. Written informed consent was obtained from all participants in the form of an electronic signature (online survey).

#### Participants

A total of 62 current faculty members were contacted and asked to take part in this study. Responses were received from 40 faculty members (64.5% response rate; 15 females, 25 males). All respondents were full-time professors representing 16 different Carnegie Type 1 (research-intensive) institutions from across the U.S. The sample of respondents included representatives from the following STEM fields (in accordance with the National Science Foundation's classification of STEM fields [12]): biology, chemistry, economics, engineering, genetics, neurobiology, physics, psychology, and sociology. All respondents had a Ph.D., and all had experience running their own research lab and training graduate students. The mean number of years since respondents received their Ph.D. was 23.5 years (SD = 11.6, minimum = 4 years, maximum = 43 years).

#### Materials

We created two videos representing two different approaches to graduate training: one approach that emphasized a total commitment to the lab and to conducting research (hereafter referred to as the *work-focused lab*), and another approach that emphasized collaboration, flexibility toward work, and accommodations for non-work needs (hereafter referred to as the *work/life-interaction-focused lab*). Each video was approximately 2 min 15 sec in length, and included a male professor/lab director (3^rd^ author) describing the expectations for lab members, and four current graduate-student members describing their lives in the lab. The *work-focused* video emphasized the traditional notions of academia: working long hours, a competitive atmosphere, and the need for single-minded dedication to one's work above all else (e.g., a graduate student in the video states: “*This lab is really competitive, but in a good way because it pushes each individual to pursue their interests farther and farther. I feel like I'm achieving more than my friends in less competitive labs.*”). In contrast, the *work/life-interaction-focused* video emphasized flexible working conditions and a collaborative atmosphere in which members were “all in it together” and had time to pursue meaningful interests outside the lab (e.g., a graduate student in the video states: “*All the students in the lab become really cohesive throughout the year. If someone is running behind getting something done at the end of the day*…*someone else is usually there to help them finish up so they're not working all night.*”). Also, the *work-focused* video depicted a predominantly male lab environment (75% male lab members) since traditionally, women were underrepresented in STEM fields and continue to be underrepresented in math-intensive fields. In contrast, the *work/life-interaction-focused* video showed a gender-balanced lab environment (50% female-50% male) that reflects a key national goal of future STEM recruitment and training.

#### Procedure

Faculty members were contacted via email and asked to participate in an online survey made available using the Qualtrics Web Survey Tool. Each respondent to the survey rated the two lab recruitment videos according to his or her personal experiences as a graduate student and as a lab director. The two videos were simply labeled *Video 1* and *Video 2*, and each respondent was asked to rate a) how much each lab resembled his or her own graduate training experience (i.e., *How they were trained*), and b) how much each lab resembled the way he or she trains students today (i.e., *How they train their students*). Respondents provided ratings based on a five-point scale, from 1 = *Not at all*, to 5 = *Exactly*. Each respondent watched one video and rated the two response items, then watched the second video and rated the two response items again. Two versions of the Qualtrics survey were created to counterbalance the presentation order of the two videos, and these two versions were distributed equally amongst the 62 faculty members who were contacted to participate. Amongst respondents, 21 watched the *work-focused lab* video first, and 19 watched the *work/life-interaction-focused lab* video first. Data are archived and accessible at: ciws.cornell.edu.

## Results

Each respondent provided ratings for four items in total: one *how they were trained* rating and one *how they train students* rating for each of the two videos. (See Table 1 for the means and standard deviations for each response item per video.) We ran a set of Pearson correlations (*r*) that included five variables: each of the four response items, and the number of years since Ph.D. (Table 2). First, we evaluated the extent to which the ratings of the two videos co-varied. We hypothesized the ratings of the two videos would be inversely correlated, thus indicating that the underlying concepts are distinct from one another. As expected, we found significant inverse correlations between the resemblance of *how they were trained* to the *work-focused lab*, and to the *work/life-interaction-focused lab*, *r* = −.60, *p*<.001; as well as between the resemblance of *how they train students* to the *work-focused lab*, and to the *work/life-interaction-focused lab*, *r* = −.42, *p* = .007 (Table 2). These results indicate that the two approaches to graduate training depicted in the videos represent approaches used in real-world labs, and that the two approaches are conceptually distinct.

**Table 1:** Study 1 Means and Standard Deviations (*SD*) of Faculty Members' Video Ratings.

Response Item	Video Type	Mean (*SD*)
How faculty were trained	Work-focused	3.13 (1.27)
	Work/life-interaction	2.90 (1.13)
How faculty train students	Work-focused	2.55 (0.99)
	Work/life-interaction	3.65 (0.95)

Note: Study 1 faculty respondents (*N* = 40) were asked to watch a *work-focused lab* video (“Work-focused”) and a *work/life-interaction-focused lab* video (“Work/life-interaction”) and rate the degree to which each video resembles (a) how they were trained as graduate students (“How faculty were trained”) and (b) how they train their own graduate students today (“How faculty train students”). Ratings were based on a scale of 1 = *Not at all* to 5 = *Exactly*.

**Table 2:** Study 1 Pearson Correlations (*r*) of Faculty Members' Video Ratings and Number of Years Since Ph.D.

	Variable	1	2	3	4
1	How faculty were trained – Work-focused	1.00	–	–	–
2	How faculty were trained – Work/life-interaction	***−.602***	1.00	–	–
3	How faculty train students – Work-focused	**.** ***540***	***−.434***	1.00	–
4	How faculty train students – Work/life-interaction	**−.326**	**.** ***614***	***−.419***	1.00
5	Number of Years since Ph.D.	.067	−.143	−.030	−.123

Note: A set of Pearson correlations were conducted that included five variables including two response items per each of the two videos, and the number of years since Ph.D. Study 1 faculty respondents (*N* = 40) were asked to watch a *work-focused lab* video (“Work-focused”) and a *work/life-interaction-focused lab* video (“Work/life-interaction”) and rate the degree to which each video resembles (a) how they were trained as graduate students (“How faculty were trained”) and (b) how they train their own graduate students today (“How faculty train students”), for a total of four response items per respondent. Ratings were based on a scale of 1 = *Not at all* to 5 = *Exactly*. “Number of years since Ph.D.” is the number of years since the respondent received his or her Ph.D. at the time the survey was completed. **Boldface** indicates significance at *p*<.05. ***Boldface italic*** indicates significance at *p*<.01.

**Table 2.** Study 1 Pearson Correlations (*r*) of Faculty Members' Video Ratings and Number of Years Since Ph.D.

Next, we evaluated the developmental impact of the type of graduate training respondents had experienced-in other words, whether the way a faculty member was trained as a graduate student influences how he or she trains students today. We found strong support for this hypothesis, with positive correlations between the resemblance of *how they were trained* and *how they train students* to the *work-focused lab*, *r* = .54, *p*<.001; and between the resemblance of *how they were trained* and *how they train students* to the *work/life-interaction-focused lab*, *r* = .61, *p*<.001 (Table 2). In addition, we found significant inverse correlations between the resemblance of *how they were trained* to the *work-focused lab*, and the resemblance of *how they train students* to the *work/life-interaction-focused lab*, *r* = −.33, *p* = .04; and between the resemblance of *how they were trained* to the *work/life-interaction-focused lab*, and the resemblance of *how they train students* to the *work-focused lab*, *r* = −.43, *p* = .005 (Table 2).

Thus, lab directors who were trained using the *work-focused* approach are far more likely to train their own students using the *work-focused* approach. Likewise, those who were trained using the *work/life-interaction-focused* approach are more likely to train their own students using the *work/life-interaction-focused* approach. In sum, the results show that the two approaches are conceptually distinct (i.e., inversely correlated), and that the type of training a student receives is predictive of how he or she later trains their own students.

Given these results, we ran an exploratory analysis to determine whether a shift away from the traditional, work-focused training methods and toward more work/life-interaction-focused training methods could be detected. Specifically, we compared the correlation between the resemblance of *how they were trained* to the *work-focused lab*, and the resemblance of *how they train students* to the *work/life-interaction-focused lab*, *r* = −.33, to the correlation between the resemblance of *how they were trained* to the *work/life-interaction-focused lab*, and the resemblance of *how they train students* to the *work-focused lab*, *r* = −.43; i.e., whether it is more likely that faculty trained with the work-focused approach train their students using the work/life-interaction-focused approach, than it is that faculty trained with the work/life-interaction-focused approach train their students using the work-focused approach. The analysis was conducted using a modified version of the Pearson-Filon statistic that incorporates Fisher's *r*-to-*z* transformation (*ZPF*). The result indicated that the difference between the two correlations was not significant, *ZPF* = .65, *p* = .26.

Since all the Pearson correlation coefficients between the four response items were significant, we conducted further analyses to compare the ratings of male and female respondents. We first calculated Pearson correlation coefficients between the four response items independently for males and females. Using Fisher's *r*-to -*z* transformation, we compared males and females on each correlation coefficient, and found no significant differences. Additionally, the number of years since Ph.D. was not significantly correlated with any of the four response items (Table 2).

## Study 2

Given the results of Study 1, we examined whether current and prospective graduate students are differentially attracted to one type of lab over the other. Based on previous survey research [4], [7], we hypothesized that current and prospective graduate students in STEM fields would prefer to join labs that incorporate work/life-interaction more so than traditional work-focused labs. Specifically, we predicted that participants (both male and female) who watch the *work/life-interaction-focused lab* video would anticipate a greater *desire to participate* in and *sense of belonging* to the lab, relative to those who watch the *work-focused lab* video.

### Method

#### Ethics Statement

Cornell University's Institutional Review Board for Human Participants approved the protocol for this study. Written informed consent was obtained from all student participants at Time 1 in the form of an electronic signature (online survey). Student participants who took part in the Time 2 experiment provided written informed consent a second time in person.

#### Participants

A total of 102 senior-year undergraduate and first- and second-year graduate students in STEM fields at a large Carnegie Type 1 (research-intensive) university were recruited via a preliminary online survey (Time 1, T1). Time 1 participants received $10 cash compensation in return for their participation. Seventy-five of the 102 T1 participants exceeded the *math identification* threshold, which qualified them for participation in the lab-based experiment (i.e, Time 2, T2).

All 75 qualifying participants were invited to take part in T2, and 53 of the 75 participants agreed to do so (28 female, 25 male; mean age = 22.1 years, minimum = 21, maximum = 26). All 53 T2 participants were U.S.-born and native English speakers. The mean time interval between T1 participation and T2 participation was 38.2 days (SD = 11.6, minimum = 10, maximum = 58). The 53 T2 participants included representatives from the following 21 fields of study: aerospace engineering, applied mathematics, biological engineering, biology, biomedical engineering, chemical engineering, chemistry, chemical biology, computer science, earth and atmospheric sciences, earth systems, economics, engineering physics, human biology, information science, materials science and engineering, mathematics, mechanical engineering, physics, structural engineering, and transportation systems engineering. Time 2 participants included 28 undergraduate seniors (14 female, 14 male), and 25 first- and second-year graduate students (14 female, 11 male). Via random assignment, 26 participants were assigned to watch the *work-focused lab* recruitment video (12 undergraduates: 6 female, 6 male; 14 graduates: 8 female, 6 male), and 27 participants were assigned to watch the *work/life-interaction-focused lab* recruitment video (16 undergraduates: 8 female, 8 male; 11 graduates: 6 female, 5 male). Time 2 participants received an additional $25 cash compensation.

#### Materials

We used the two lab recruitment videos validated in Study 1: the *work-focused lab* video and the *work/life-interaction-focused lab* video. For further details, see the method section of Study 1.

#### T1 Survey

The T1 survey was available online using the Qualtrics Web Survey Tool. The survey included items from two measures: *math identification* and *commitment to science* (Table 3). Demographic information was also obtained. The *math identification* measure consisted of two statements (e.g., *I am good at math tasks*) [13]. Participants rated their level of agreement with each statement on a 7-point scale, ranging from *Not at all* to Extraordinarily. Each participant's *math identification* score was the sum of his or her two ratings. The *commitment to science* measure also consisted of two statements (e.g., *A career in a STEM field is well-suited to my particular strengths and abilities*) rated on the same 7-point scale. Each participant's *commitment to science* score was the sum of his or her two ratings. Consistent with previous research that required highly math-identified participants [13], T1 participants were required to have a *math identification* score of 10 or higher to qualify for participation at T2.

**Table 3:** Time 1 Survey Items.

**Math Identification**
*I am good at math tasks.*
*It is important to me that I do well on math tasks.*
**Commitment to Science**
*I am committed to pursuing a career in a STEM field.*
*A career in a STEM field is well suited to my particular strengths and abilities.*

Note: All questions above were presented to Study 2 participants as a single list, and did not include the measures' labels as depicted in this table. Participants were instructed as follows: *Please rate the following statements, using the following scale, 1 = Not at all, 2 = Slightly, 3 = Somewhat, 4 = Generally, 5 = Very, 6 = Extremely, 7 = Extraordinarily*. The Commitment to Science measure was also included in the Time 2 survey. Participants' score on each measure was the sum of his or her two ratings of the respective two statements of each measure.

#### T2 Experiment

Time 2 participants were not aware of the true purpose of the experiment. Each participant was simply told that he or she would be assessing the effectiveness and quality of a science lab recruitment video as part of a study to improve recruitment into STEM fields. Each participant was randomly assigned to watch one of two lab recruitment videos differing in portrayal of the lab environment: a *work-focused lab* or a *work/life-interaction-focused lab*. Each participant completed T2 individually.

After viewing the video, each participant completed a series of survey items (Table 4) that included the *commitment to science* measure from T1 (Table 3). Two questions pertained to the supposed purpose of the experiment (e.g., *How successful would you be after working in this lab?*). These questions also served as a control check on the two videos' similarity, in terms of participants' perceptions of how effective the labs are in helping student members become successful and get the jobs they seek (Table 4). Another eight questions comprised the two four-question primary measures: *sense of belonging* and *desire to participate* (Table 4) [13]. *Sense of belonging* assessed the extent to which participants would feel like they belong in the lab, if they joined (e.g., *How comfortable do you anticipate feeling in this lab?*). *Desire to participate* assessed participants' willingness to join the lab (e.g., *How likely would you be to actually join this lab?*). All questions were answered using the same 7-point scale used in the T1 survey. On both primary measures, scores were calculated for each participant by summing the ratings of the respective four statements of each measure. Also, the *commitment to science* measure was completed a second time (first completed at T1) to determine whether viewing the video altered students' plans to pursue a scientific career. Upon completion of the questionnaires, each participant was debriefed and informed of the true purpose of the experiment. Data are archived and accessible at: ciws.cornell.edu.

**Table 4:** Time 2 Survey Questions.

**Control Check**
*How successful would you be after working in this lab?*
*How much would this lab prepare you to get the job you seek?*
**Sense of Belonging**
*Do you anticipate feeling like you would belong as a member of this lab?*
*How comfortable do you anticipate feeling in this lab?*
*How much do you feel like you could “be yourself” in this lab?*
*How accepted do you think you will feel in this lab?*
**Desire to Participate**
*How interested are you in this lab after watching the video?*
*How likely would you be to actually join this lab?*
*How appealing would this lab be to the typical student?*
*How much do you want to join this lab?*

**Figure 1:**
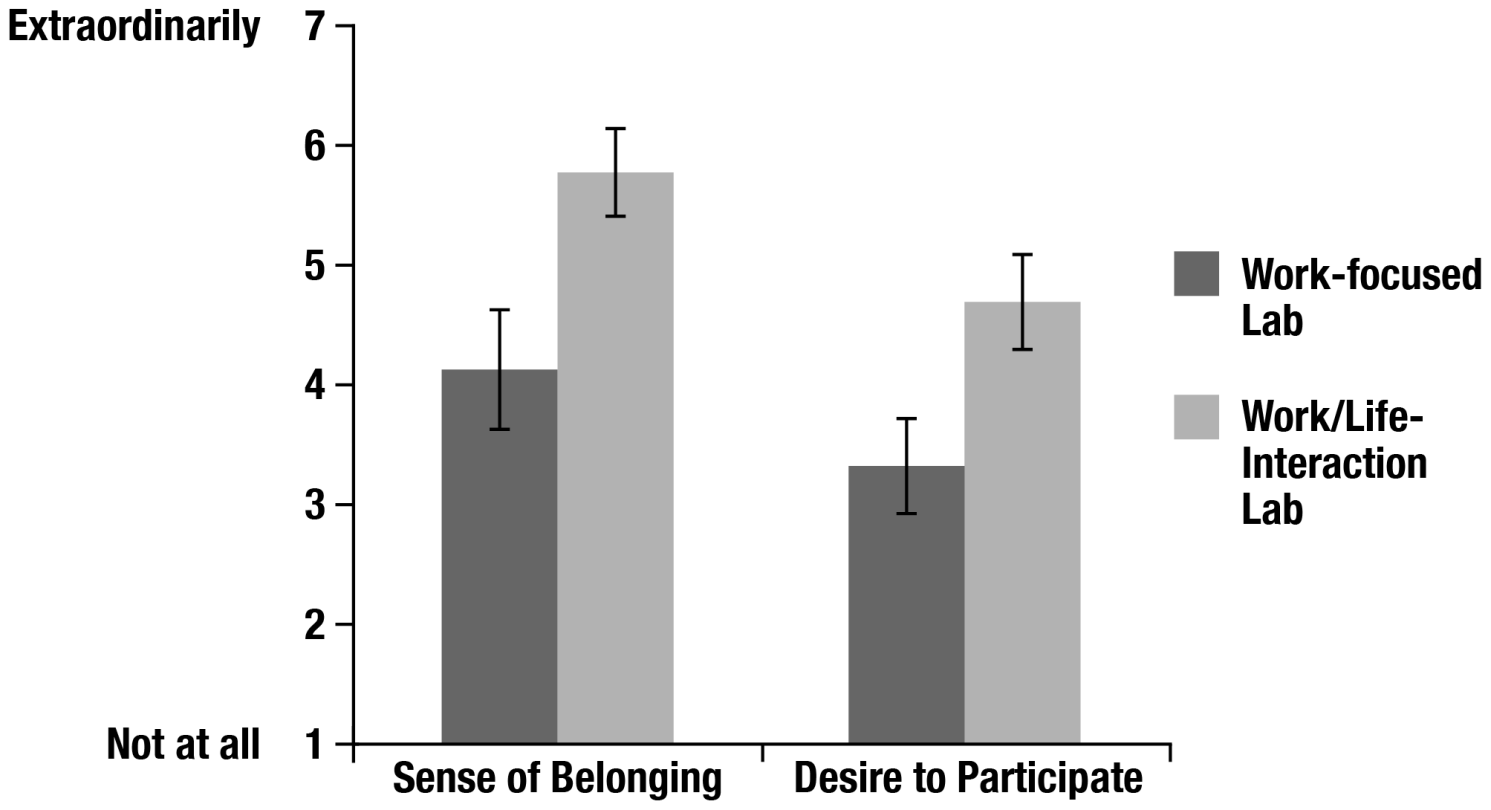
Students' *Sense of Belonging* and *Desire to Participate* Ratings by Video Type. Student participants were randomly assigned to watch either the *work-focused lab* recruitment video or the *work/life-interaction-focused lab* recruitment video. After viewing the video, each participant completed two four-question measures in which he or she rated his or her anticipated *sense of belonging* to the lab in the video if he or she were to join, and his or her *desire to participate* in the lab in the video. Ratings were based on a 7-point scale from 1 = *Not at all* to 7 = *Extraordinarily*. Bars in the figure represent the mean rating for each measure, by video. For each measure, the mean difference between the two video types was statistically significant. Error bars +/− 2 standard errors.

Note: All questions above were presented to Study 2 participants as a single list, and did not include the measures' labels as depicted in this table. Participants were instructed as follows: *Please answer the following questions about the lab depicted in the recruitment video you watched, using the following scale, 1 = Not at all, 2 = Slightly, 3 = Somewhat, 4 = Generally, 5 = Very, 6 = Extremely, 7 = Extraordinarily*. The Time 2 survey also included the Commitment to Science measure (not shown here) used in the Time 1 survey (see Table 3). Participants' score on each of the Sense of Belonging and Desire to Participate measures was the average of his or her ratings of the respective four statements of each measure (See Table 6 and Figure 1 for results).

### Results

#### Commitment to Science

*Commitment to science* was assessed at both T1 and T2, providing the means for a within-subjects pre-/post-manipulation comparison of the effect of watching the recruitment video on participants' commitment to science. A three-way univariate analysis of variance (ANOVA) was conducted with participant gender (male, female), video type (work-focused, work/life-interaction-focused), and time point (T1, T2) entered as the three fixed factors. Both participant gender and video type were between-subjects factors, and time point was a within-subjects factor. The analysis did not reveal any significant effects. Descriptively, scores increased slightly from T1 to T2, however, these differences were not statistically significant (Table 5).

**Table 5:** Study 2 Means and Standard Deviations of Commitment to Science Scores at Time 1 and Time 2, by Video Type and Participant Gender.

Video Type	Gender	*N*	Time 1	Time 2
Work-focused	Female	14	12.29 (1.59)	12.93 (1.44)
	Male	12	12.17 (1.47)	12.25 (1.91)
Work/life-interaction	Female	14	11.14 (2.18)	11.71 (1.63)
	Male	13	11.85 (3.18)	12.00 (2.48)

Note: Commitment to Science was assessed both before (Time 1, or T1) and after (Time 2, or T2) the experimental manipulation, and consisted of two statements pertaining to a commitment to pursuing a career in a STEM field. The manipulation took place in the beginning of T2, in which each participant viewed either the *work-focused lab* video (“Work-focused”) or the *work/life-interaction-focused lab* video (“Work/life-interaction”). Both before and after viewing one of the videos, each participant rated his or her level of agreement with each statement on a scale of 1 = *Not at all*, to 7 = *Extraordinarily*. Scores are the sum of each participant's two ratings per time point (T1, T2). Data in each cell of the Time 1 and Time 2 columns in this table depict the mean score with the standard deviation in parentheses.

#### Control Check

Two T2 questions were analyzed to determine the equivalency of the two videos on factors-other than the *work-focused* vs. *work/life-interaction-focused* manipulation-that could affect participants' *sense of belonging* and *desire to participate* ratings, and potentially confound the results. Each question was analyzed using an independent samples *t*-test. Participants' perception of how successful they would be after working in the lab did not differ between those who watched the *work-focused lab* video, *M* = 4.42, and those who watched the *work/life-interaction-focused lab* video, *M* = 4.59, *t*(52) = −.54, *p* = .59. Participants' perception of how well the lab would prepare them to get the job they seek also did not differ between those who watched the *work-focused lab* video, *M* = 4.15, and those who watched the *work/life-interaction-focused lab* video, *M* = 3.85, *t*(52) = .92, *p* = .36. Thus, any observed differences between the two lab types on the primary measures cannot be attributed to one lab being perceived as a better source of preparation for a science career than the other-both the *work-focused lab* and the *work/life-interaction-focused lab* were perceived as good sources of preparation.

#### Sense of Belonging

*Sense of belonging* scores were analyzed in a three-way univariate ANOVA with participant gender (male, female), video type (work-focused, work/life-interaction-focused), and student status (undergraduate, graduate) entered as the three, between-subjects fixed factors. (See Table 6 for a full breakdown of the *sense of belonging* means and standard deviations.) This analysis revealed only one significant effect: a large main effect of video type, *F*(1, 45) = 26.0, *p*<.001, Cohen's *d* = 1.49 (Figure 1). We conducted follow-up contrasts within each of the other two factors (participant gender and student status) to further examine this main effect. All follow-up contrasts were conducted using independent samples *t*-tests, and all were statistically significant. Within-gender follow-up contrasts confirmed that both female participants and male participants who watched the *work/life-interaction-focused lab* video reported a greater *sense of belonging* to the lab relative to their same-sex counterparts who watched the *work-focused lab* video, *t*(45) = 4.50, *p*<.001, and *t*(45) = 2.77, *p* = .008, respectively. Within-status follow-up contrasts confirmed that both undergraduate participants and graduate participants who watched the *work/life-interaction-focused lab* video reported a greater *sense of belonging* to the lab relative to their same-status counterparts who watched the *work-focused lab* video, *t*(45) = 3.51, *p* = .001, and *t*(45) = 3.70, *p* = .001, respectively.

**Table 6:** Study 2 Means and Standard Deviations for Sense of Belonging (SB) and Desire to Participate (DP), by Video Type, Participant Gender, and Student Status.

Video Type	Gender	Status	*N*	SB	DP
Work-focused	Female	Undergraduate	6	2.92 (1.19)	2.67 (1.02)
		Graduate	8	2.94 (1.33)	2.03 (0.89)
		Total	14	2.93 (1.23)	2.30 (0.97)
	Male	Undergraduate	6	3.88 (1.25)	2.71 (1.29)
		Graduate	6	2.83 (1.27)	2.00 (0.99)
		Total	12	3.35 (1.32)	2.35 (1.16)
	Total	Undergraduate	12	3.39 (1.27)	2.69 (1.11)
		Graduate	14	2.89 (1.26)	2.02 (0.90)
		Total	26	3.13 (1.26)	2.33 (1.04)
Work/life-interaction	Female	Undergraduate	8	5.12 (0.94)	3.81 (1.29)
		Graduate	6	4.63 (0.70)	4.00 (0.97)
		Total	14	4.91 (0.86)	3.89 (1.13)
	Male	Undergraduate	8	4.70 (1.33)	3.25 (0.96)
		Graduate	5	4.55 (0.57)	3.90 (0.72)
		Total	13	4.64 (1.07)	3.50 (0.91)
	Total	Undergraduate	16	4.91 (1.13)	3.53 (1.14)
		Graduate	11	4.59 (0.62)	3.95 (0.83)
		Total	27	4.78 (0.96)	3.70 (1.03)

Note: The Sense of Belonging (SB) and Desire to Participate (DP) measures each consisted of four statements, and gauged participants' anticipated sense of belonging to and desire to participate in the lab depicted in the video. Prior to these measures, participants watched either the *work-focused lab* video (“Work-focused”) or the *work/life-interaction-focused lab* video (“Work/life-interaction”). Participants rated each statement on a scale of 1 = *Not at all*, to 7 = *Extraordinarily*, and a participant's score on each measure was the mean of his or her ratings of the four respective statements of each measure. “Student Status” refers to the current status of the participant as either an undergraduate or graduate student. Data in each cell of the *SB* and *DP* columns in this table depict the mean score with the standard deviation in parentheses.

#### Desire to Participate

*Desire to participate* scores were analyzed in a three-way univariate ANOVA with participant gender (male, female), video type (work-focused, work/life-interaction-focused), and student status (undergraduate, graduate) entered as the three, between-subjects fixed factors. (See Table 6 for a full breakdown of the *desire to participate* means and standard deviations.) This analysis also revealed only one significant effect: a large main effect of video type, *F*(1, 45) = 22.8, *p*<.001, *d* = 1.33 (Figure 1). We conducted follow-up contrasts within each of the other two factors (participant gender and student status) in order to further examine this main effect. All follow-up contrasts were conducted using independent samples *t*-tests, and all were statistically significant. Within-gender follow-up contrasts confirmed that both female participants and male participants who watched the *work/life-interaction-focused lab* video reported a greater *desire to participate* in the lab relative to their same-sex counterparts who watched the *work-focused lab* video, *t*(45) = 3.91, *p*<.001, and *t*(45) = 2.88, *p* = .006, respectively. Within-status follow-up contrasts confirmed that both undergraduate participants and graduate participants who watched the *work/life-interaction-focused lab* video reported a greater *desire to participate* relative to their same-status counterparts who watched the *work-focused lab* video, *t*(45) = 2.12, *p* = .04, and *t*(45) = 4.57, *p*<.001, respectively.

## Discussion

The results of Study 1 showed that the approaches to graduate training depicted in the *work-focused* and *work/life-interaction-focused* videos are conceptually distinct approaches used by current lab directors in real-world labs. As seen, the type of labs that current lab directors were trained in has a long reach, influencing the kind of lab experience they create for their own students today. The results of Study 2 suggest that current and prospective STEM graduate students with high levels of math identification are less interested in joining science labs portrayed in traditional ways, than they are in joining labs with more work/life-interaction. Participants who watched the *work/life-interaction-focused lab* video reported a greater *sense of belonging* to and *desire to participate* in the lab depicted in the video, compared to participants who watched the *work-focused lab* video. Both effects were quite large, and occurred despite the fact that participants rated both labs similarly in terms of how successful they would be after working in the lab and how well the lab would prepare them to get the job they seek. In addition, neither video had any effect on participants' *commitment to science*: Participants in both conditions were strongly committed to a career in science, both before and after the experimental manipulation.

These findings suggest that senior scientists who endorse the attitudes and goals of the *work-focused lab* could lose talent to other labs, provided that *work/life-interaction-focused labs* are an option. Moreover, if the traditional, work-focused approach is the only option available, students could induce that it is the only way labs are run, and science in general could lose talent to non-scientific careers that are better adapted to Millennials' desire for work/life-interaction. And, as was found in Study 1, one generation's approach to graduate training can have a lasting impact, given that scientists are likely to train graduate students in a manner similar to the way they themselves were trained.

These studies support the view that Millennials entering graduate school in STEM fields, such as the participants in Study 2, seek environments characterized by work/life-interaction [4], [7], and show that this preference exists independently of how well these students believe the labs prepare them for later careers. Once hailed as primarily a woman's desire [2], [10], [11], our findings reveal that the preference for work/life-interaction is now shared by men, a point recently observed across many fields, including STEM, medicine, and law [14]. In addition to changing workforce demographics, this shift in attitudes and preferences toward work/life-interaction may also relate to changing norms for division of household labor among educated couples [10], [11]. Time for family, travel, and relaxation are more important to both female and male Millennials than was true of previous generations [4], [14], [15]. Traditional sources of advice for graduate students such as *The Compleat Academic* [16]-that recommend a consuming, linear approach to graduate training-may not appeal to many of today's students. Millennials have different expectations than members of the “Boomer” generation who now run most labs [2], and consequently, the latter should be aware of this cultural shift. The problem of reaching and attracting the best talent in the current cohort of students may require a new approach, and raises intriguing issues regarding feasibility and productivity that, although beyond the scope of this experiment, should be discussed by members of professional scientific associations.

It may be easier to accommodate Millennials' desire for work/life-interaction in some fields than in others in which the demands for stringent lab hours can be crucial for the conduct of research. The philosopher Sommers [17], responding to women's demand for greater work/family-interaction, noted that some have labeled traditional male scientists' work habits as representing obsessive, single-minded dedication, with an “intense desire for achievement” that some allege not only marginalizes women, but also may compromise good science. Paraphrasing one gender activist, Sommers reports, “If we continue to emphasize and reward always being on the job, we will never find out whether leading a balanced life leads to equally good or better scientific work” [17]. She is, however, critical of this view noting that, “A world where women (and re-socialized men) earn Nobel Prizes on flextime has no relation to reality” [17]. We take no position in this debate beyond noting that perhaps in some fields it will be difficult and/or undesirable to deemphasize the “obsessive” work habits and single-minded dedication that characterize traditional, work-focused labs. We leave it to scientists and their professional societies to determine whether the desire of today's talented students for greater work/life-interaction can be accommodated within the strictures of their graduate training models without compromising scientific progress.
